# Letters

**Published:** 1910-01

**Authors:** 


### Letters.

McDade, Texas, December 17, 1909.

*Dr. F. E. Daniel:*

Dear Doctor: Here is a green leaf to run us till June, 1910, and to help the mascot. My best wishes to you and wife and a Happy Christmas. Long live the "Red Back," Daniel and Lydston. I have no patience with Simmons.

Yours truly,

G. W. Southern.

---

Winnsboro, Texas, December 20, 1909.

*Dr. F. E. Daniel:*

Dear Sir:—Peace find $1.00 for "Red Back." I think I am one of your *oldest subscribers.* I have been taking the "Red Back" regularly ever since first issue. I am the oldest practitioner in Wood county, and have been "regular" in the harness forty years, and am still able to work all the time. Don't know what else to do, so can't retire. Wishing you a Merry and Happy Christmas, and may you continue to be useful in the future as you have been in the past.

Respectfully,

T. N. Skeen.

PEARSALL, TEXAS, December 11, 1909.

*Dr. F. E. Daniel, Austin, Texas.*

DEAR DOCTOR: Enclosed please find check for one dollar for one year to the Texas "Red Back." The "Red Back" is the livest wire in the State or any other State in the way of a medical journal, so please keep it coming.

And here is hoping that "Bettie and the baby" have a Xmas and that the doctor lives for many years to edit the "Red Back."

Yours very truly,

HOUSTON NEELEY.

---

GREENVILLE, TEXAS.

*Dr. F. E. Daniel, Austin, Texas.*

MY DEAR DOCTOR: Please accept postoffice money order for $1.00 for another year's subscription to the "Red Back." I must have it to keep me in remembrance of the dearly loved old ethics now fast departing from our profession under the new regime which if followed will make us tradesmen rather than members of a once noble profession. I wish you a Merry Christmas and a prosperous New Year.

Fraternally,

T. J. MILNER.

---

RICHLAND SPRINGS, TEXAS, December 7, 1909.

*Dr. F. E. Daniel, Austin, Texas.*

DEAR DOCTOR: I hereby hand you check for one year's subscription to the "Red Back." Can't afford to miss a copy. Just notify me when I am in arrears and I'll endeavor to "do it now" every time. I admire your loyalty to old-time, high-toned medical ethics and the way you handle some subjects "without gloves."

Yours sincerely,

A. D. NELSON.

---

CHIHUAHUA, MEXICO, December 16, 1909.

*Dr. F. E. Daniel, Editor of the "Red Back."*

DEAR DOCTOR: I have been a subscriber to your interesting, independent, little journal for about two years and I have enjoyed reading it very much, so much so that I should like to continue my subscription for another year. I have not yet paid my subscription for the current year, so I enclose a draft for $2.00, which I trust will be satisfactory.

Wishing you success and a Merry Xmas,

Yours fraternally,

H. D. EATON,

P. O. Box 11. Chihuahua, Mexico.

